# Depression in Cardiac Patients Is a Major Cardiovascular Event Risk Factor: A 12-Month Observational Study

**DOI:** 10.3390/jcm13226911

**Published:** 2024-11-16

**Authors:** Jakub Podolec, Paweł Kleczyński, Marcin Piechocki, Michał Okarski, Katarzyna Lizończyk, Kornelia Szkodoń, Andrzej Silczuk, Tadeusz Przewłocki, Jacek Legutko, Anna Kabłak-Ziembicka

**Affiliations:** 1 Department of Interventional Cardiology, Institute of Cardiology, Jagiellonian University Medical College, 31-007 Kraków, Poland; jakub.podolec@uj.edu.pl (J.P.); kleczu@interia.pl (P.K.); jacek.legutko@uj.edu.pl (J.L.); 2 Department of Interventional Cardiology, The St. John Paul II Hospital, 31-202 Kraków, Poland; michal.okarski@alumni.uj.edu.pl (M.O.); tadeuszprzewlocki@op.pl (T.P.); 3 Department of Cardiac and Vascular Diseases, Institute of Cardiology, Jagiellonian University Medical College, 31-007 Kraków, Poland; mpiech98@gmail.com; 4 Department of Vascular and Endovascular Surgery, The St. John Paul II Hospital, 31-202 Kraków, Poland; 5 Doctorial School of Medical and Health Sciences, Jagiellonian University Medical College, 31-007 Kraków, Poland; 6 Students’ Scientific Group of Modern Cardiac Therapy, Department of Interventional Cardiology, Institute of Cardiology, Jagiellonian University Medical College, 31-007 Kraków, Poland; katarzyna.lizonczyk@gmail.com (K.L.); kornelia.szkodon@gmail.com (K.S.); 7 Department of Environmental Psychiatry, Faculty of Life Sciences, Medical University of Warsaw, 02-091 Warsaw, Poland; asilczuk@ipin.edu.pl; 8 Noninvasive Cardiovascular Laboratory, The St. John Paul II Hospital, Prądnicka 80, 31-202 Kraków, Poland

**Keywords:** acute coronary syndrome, acute heart failure, aortic valve stenosis, Beck Depression Inventory, cardiovascular disease, chronic coronary artery disease, depression, Hamilton Depression Rating Scale, major cardiac and cerebrovascular events

## Abstract

**Background**: Depression is a known factor in poor cardiovascular outcomes but is often underassessed in cardiac units. This study evaluates the impact of depression on cardiovascular outcomes in patients undergoing cardiac interventions. **Methods:** The study included 133 patients who underwent uncomplicated procedures for degenerative aortic valve stenosis (n = 40), acute coronary syndrome (n = 29), or chronic coronary artery disease (n = 64). Depression was assessed using the Beck Depression Inventory (BDI) and Hamilton Depression Rating Scale (HAM-D). The primary endpoint was a major adverse cardiac and cerebrovascular event (MACCE). Patients were followed up for 12 months. Cox proportional hazards analysis was used to identify MACCE risk factors. **Results**: Depression was more frequently screened by HAM-D than BDI (42.9% vs. 30.8%, *p* < 0.001). During follow-up, 26 (19.5%) MACCEs occurred. In univariate analysis, risk factors included BDI score ≥ 11, HAM-D score ≥ 8, diabetes on insulin, anticoagulant use, atrial fibrillation, and serum creatinine level ≥ 130 µmol/L. Depression in the BDI increased the risk of the MACCE 3.6-fold (95%CI: 1.64–8.0, *p* = 0.001), whereas in the HAM-D, it increased the risk 4.9-fold (95%CI: 1.97–12.24, *p* < 0.001). Multivariate analysis showed HAM-D score ≥ 8 as the strongest predictor of MACCE (HR: 3.08, 95%CI: 1.18–8.08). **Conclusions**: Depression is a common finding in cardiovascular patients, and it is a strong risk factor for one-year cardiovascular mortality and adverse event risk. Therefore, we believe that common guidelines should be elaborated between relevant psychiatry and cardiology scientific societies.

## 1. Introduction

There is a common agreement that depressive symptoms influence poorer cardiovascular outcomes [1,2]. However, little attention is paid to revealing and grading depression in cardiovascular patients in most cardiac units [3,4]. This can be partially explained by the lack of sufficient evidence in the literature that treatment of depressive symptoms alters cardiovascular outcomes [5,6]. Furthermore, there is no single test for depression, whereas depression scales are numerous [7,8,9,10,11,12]. Lastly, psychiatric scientific councils lack consensus on the specific depression test(s) that should be used for patients with cardiovascular disease [13,14,15].

Patients with cardiovascular disease, particularly those referred for urgent or prescheduled cardiovascular invasive interventions, constitute a particularly vulnerable subset of patients [16,17,18,19,20,21]. Cardiovascular patients may experience anxiety, fatigue, poor concentration, and sleep disorders at considerable levels [22,23]. They may feel anxious about unknown (vicious, hostile) environments and medical staff, hospital stays, the invasive procedure itself, procedure complications, and further outcomes. Cardiac patients usually suffer from many cardiovascular comorbidities, including diabetes, arterial hypertension, lipid disorders, chronic heart failure, diastolic dysfunction, and many concomitant disorders such as respiratory tract, orthopedic, and neurological disorders, which require the use of many medications [24,25,26,27]. The increasing number of comorbidities in cardiac patients is mostly dependent on the aging population phenomenon [28,29]. Lastly, diminished mood resulting from multi-morbidity contributes to the development of depression and dementia.

Therefore, in the present study, we have not only analyzed the prevalence of depression among patients referred for complex and difficult cardiac interventional therapies, but also compared the utility of two widely used psychiatric assessment scales: the Beck Depression Inventory (BDI) and the Hamilton Depression Rating (HAM-D). This is highly relevant, as despite reviews and observational studies supported by the guidelines of scientific societies recommending identification of depression in cardiovascular patients, there is no agreement on the specific single test that should be used [30].

The Hamilton Depression Rating Scale (HAM-D) is the longest-standing test for assessing the likelihood of depression, and with an established position for several decades [9]. However, the Beck Depression Inventory (BDI) has also been utilized for many years, offering a more straightforward alternative to the HAM-D. Its advantages include self-administration by patients and a shorter completion time [8,31].

Therefore, in this study, we have attempted to (1) assess cardiovascular outcomes regarding depression incidence and severity during a 12-month follow-up, in patients who underwent cardiovascular intervention in the Cardiac Unit; and (2) compare associations between scores obtained in the BDI and HAM-D scales with cardiovascular outcomes.

## 2. Materials and Methods

### 2.1. Study Population

**Figure 1:**
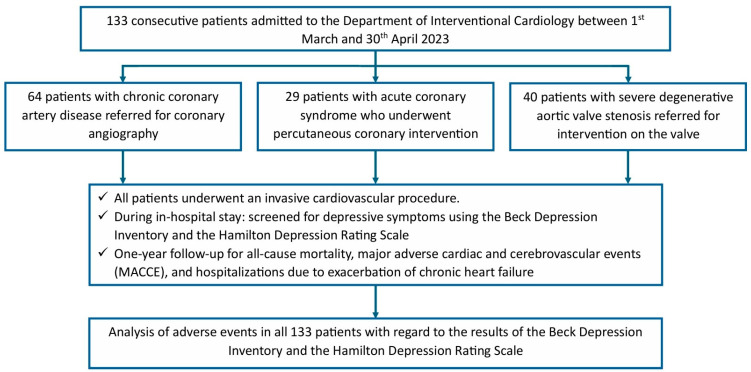
Study flow chart.

A total of 133 consecutive patients with cardiovascular disease, with a median age of 71 years (Q1–Q3: 65–75) were included following uncomplicated intervention on severe degenerative aortic valve stenosis (DAS, n = 40), acute coronary syndrome (ACS, n = 29), or chronic coronary artery disease (CCAD, n = 64) between the 1 March 2023 and 30 April 2023 in the Cardiac Unit. The study flow chart is presented in Figure 1.

The inclusion criteria included age between 18 and 90 years, signed informed consent, and hospitalization for one of the following conditions: CCAD (ICD-10 codes: I20, I25), ACS (I21, I22, I24), or DAS (I35.0, I35.2). The exclusion criteria were not meeting the conditions for inclusion, history of dementia, stroke, or transient ischemic attack (no neurological symptoms present or past, and/or ischemic lesions on brain CT scans confirming the cerebral ischemia as ensured by the consulting neurologist), hemodynamic instability, active cancer or history of valve/cardiac surgery, and lack of informed consent and inability to obtain informed consent due impaired consciousness. On admission, all patients underwent evaluation for cardiovascular risk factors and the biochemical blood samples for lipids, renal function, thyroid stimulating hormone (TSH), and glucose levels. All patients underwent assessment for depressive symptoms using the BDI and the HAM-D scales. Patients with a result indicating depression were instructed at discharge to visit a mental health outpatient clinic.

The study protocol was consistent with the requirements of the Helsinki Declaration and was approved by the Institutional Ethics Committee of the Jagiellonian University (KBET/1072.6120.148.2018). All patients signed the informed consent to participate in the study.

### 2.2. Tests Used for Depression Assessment

During index hospitalization, each patient was assessed for depressive symptoms using two questionnaires: the Beck Depression Inventory (BDI) and the Hamilton Depression Rating Scale (HAM-D). BDI as the self-assessment screening tool was completed by patients, whereas the HAM-D score was obtained through an interview conducted by 1 of 4 doctors who were experienced and well trained in the diagnosis of depression. The probable diagnosis of depression was initially based on the BDI and HAM-D scales. In case of a positive score for depression, patients were referred to psychiatrists to confirm the diagnosis of depression, and afterwards were given antidepressants if needed.

BDI is a 21-item, self-report rating inventory that measures the characteristic attitudes and symptoms of depression. Each item is rated on a 4-point scale ranging from 0 to 3, based on the severity in the last two weeks. The questionnaire is commonly self-administered although initially designed to be administered by trained interviewers. The results of the BDI score were interpreted as follows: 0–11 points—no depressive symptoms, 12–26 points—mild depression, 27–49 points—moderate depression, and 50–63 points—severe depression.

The HAM-D is a clinician-based questionnaire that consists of 17 elements, measuring the severity of depressive symptoms. The interviewer rates the level of agitation or how the symptoms impact the patient’s everyday life. Each item is scored on a basic numeric scoring of 0 to 4 points: a score >7/52 is taken to indicate depression. For HAM-D, the following scoring scale was used: 0–7 points—no depressive symptoms, 8–12 points—mild depression, 13–17 points—moderate depression, 18–29 points—severe depression, and 30–52 points—very severe depression. There is also 24-item HAM-D, which incorporates components for evaluating somatic symptoms. Given that the study population presented with somatic complaints attributable to etiologies other than depression, we decided to employ the 17-item version for a more adequate assessment.

### 2.3. Follow-Up Period

During an observation period of 12 months, the incidence of all-cause death, cardiovascular death (CVD), myocardial infarction, ischemic stroke, hospitalization for acute heart failure (AHF), and dementia were recorded. The composite endpoint were major adverse cardiac and cerebrovascular events (MACCEs) defined as all-cause death, non-fatal myocardial infarction, ischemic stroke, and hospitalization for AHF.

CVD was defined as fatal (ischemic stroke, myocardial infarction, or acute heart failure episode), or other CVD (i.e., any sudden or unexpected death unless proven as non-cardiovascular on autopsy). AHF episodes were defined as hospitalization for newly diagnosed exacerbated heart failure requiring administration of intravenous diuretics and/or vasoactive drugs (dopamine, dobutamine, epinephrine, or norepinephrine).

The final follow-up visit was performed as a telephone visit with the patient or mandated family member. For all patients, data regarding patient vital status were obtained from the national health registry at the time of database closure.

### 2.4. Statistical Analysis

Standard descriptive statistics were used to describe the data. Qualitative data are presented as numbers with percentages [n (%)], and groups were compared using the Pearson chi-square test, Fisher test, Fisher—Freeman–Halton test, and McNemar test. The normality of the distribution of quantitative data was investigated using the Shapiro–Wilk test. Due to non-normal distribution, all quantitative data are shown as the median with quartiles 1 and 3 [median (Q1–Q3)] and compared using the U Mann–Whitney test for 2 groups or the Kruskal–Wallis test with post hoc Dunn’s test for more than 2 groups. Associations between the quantitative variables and BDI and HAM-D scores were assessed using Spearman’s rank correlation coefficient and are presented as rho with a 95% confidence interval. For quantitative data, to establish the best cutoff values that differentiate patients in terms of the occurrence of a composite endpoint, receiver operating characteristic (ROC) curves with the area under the ROC curve (AUC) were determined. Sensitivity, specificity, and accuracy were calculated for the optimal cutoff value, which was identified using Youden’s J statistic. Composite-endpoint-free survival curves were constructed using the Kaplan–Meier estimator, and group comparisons were made using the log-rank test. The univariate Cox proportional hazards analysis was performed to determine the risk factors for a composite event. A re-analysis was performed for quantitative variables with a *p*-value of less than 0.05 in univariate COX analysis, treating the variable as binary with the cutoff point determined by ROC analysis. For all parameters with a *p*-value less than 0.05, a multivariate Cox proportional hazards model was created using the enter method. The significance level α was set at 0.05. All statistical analyses were carried out using PS IMAGO PRO 9.0.

## 3. Results

### 3.1. Baseline Patients’ Characteristics

A total of 133 consecutive patients admitted with a diagnosis of DAS, CCAD, or ACS were included into this study. A BDI score of 12 or more points, suggesting depression, was obtained in 41 (30.8%) of study participants, whereas this number increased to 57 (42.9%) patients on the HAM-D scale (with a score of seven or more points). Among these patients, 30 had results beyond the norm in both questionnaires, while 11 patients had elevated BDI scores only and 27 patients had elevated HAM-D scores only [*p* < 0.001]. Interestingly, the BDI depression scores did not differ with respect to the presence and severity of cardiovascular risk factors, nor the incidence of respiratory tract, thyroid gland, or atrial arrhythmia comorbidities. In contrast, the HAM-D score, suggestive of depression, was more frequently obtained in older patients suffering from diabetes, atrial fibrillation, on oral anticoagulants, and those with lower eGFR (Table 1). Lifetime smoking habits, thyroid gland disease, chronic obstructive pulmonary disease (COPD), and asthma did not differ between patients who obtained HAM-D scores indicative of depression and those with normal scores. Antidepressants were taken by 15 (11.3%) of the study participants at discharge.

### 3.2. Associations Between Depression Severity and Cardiovascular Risk Factors

Age was weakly positively correlated with the BDI score (Spearman’s rho: 0.24, 95%CI: 0.07–0.4, *p* = 0.006) and HAM-D score (rho: 0.32, 95%CI: 0.16–0.47, *p* < 0.001). A weak negative correlation was observed between eGFR and HAM-D scores (rho: −0.22, 95%CI: (−0.38)–(−0.044), *p* = 0.012). The results of both questionnaires were strongly positively correlated (rho: 0.63, 95%CI: 0.52–0.73, *p* < 0.001). The other quantitative parameters assessed in the study were not significantly correlated with both BDI and HAM-D scores.

Details about patients’ characteristics by severity of depression in BDI and HAM-D are shown in Table 2.

### 3.3. All-Cause Death and Cardiovascular Outcomes

During a follow-up period of 12 months, nine (6.8%) patients of the 133 study participants died. None of the patients died by suicide. All-cause death occurred in seven (10.9%) patients in the CCAD group, two (5%) in the DAS group and none in the ACS group (*p* = 0.13). Depression severity was an important risk factor of all-cause death in the HAM-D score, increasing from 2.6% in patients without depressive symptoms, to 9.4% in mild depression, 12.5% in moderate, and 22% in severe depression (*p* = 0.036). Depression severity was also an important risk factor of MACCE, increasing from 7.9% in patients without depressive symptoms, to 31.3% in mild depression, 37.5% in moderate, and 44.4% in severe depression (*p* < 0.001).

MACCEs were recorded in twenty-six (19.5%) patients, including death in nine (6.8%), myocardial infarction in two (1.5%), ischemic stroke in four (3%), and hospitalization for AHF in twelve (9%) patients. Some patients suffered from multiple cardiovascular events. Patients with depression in BDI and HAM-D, compared to patients with no depressive symptoms, had a 2.6-fold and a 4.4-fold higher incidence of a MACCE (*p* = 0.008 and *p* < 0.001), respectively (Table 3). Furthermore, three patients developed dementia during a follow-up period.

### 3.4. Factors Affecting Adverse Cardiovascular Outcomes

In the ROC analysis, the BDI and HAM-D demonstrated significant AUC values. The optimal cutoff points for predicting MACCE were identified as BDI ≥ 11 and HAM-D ≥ 8. The AUC for the other variables did not significantly differ from 0.5 (Table 4).

**Figure 2:**
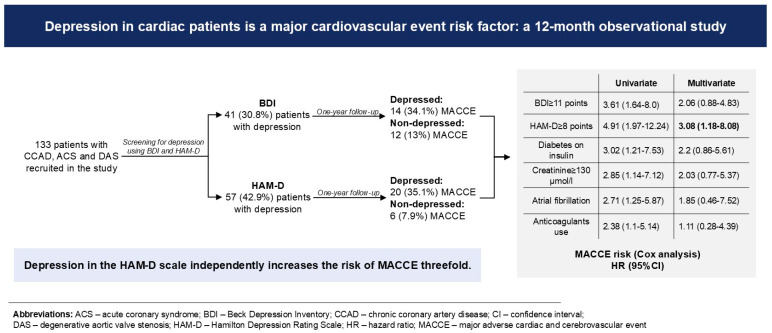
Association between results of the BDI and HAM-D scores and one-year cardiovascular outcomes.

In a univariate Cox proportional hazard ratio analysis, the presence of depression on the HAM-D scale (HR: 4.91, 95% CI: 1.97–12.24, *p* < 0.001), a BDI score ≥ 11 points (3.61, 1.64–8.0, 0.001), diabetes on insulin (3.02, 1.21–7.53, 0.018), use anticoagulants, either NOAC or VKA (2.38, 1.10–5.14, 0.028), atrial fibrillation (2.71, 1.25–5.87, *p* = 0.011), and creatinine level ≥ 130 µmol/L (2.86, 1.15–7.12, *p* = 0.024) showed associations with a MACCE risk (Table 5, Figure 2).

In the multivariate Cox proportional hazard analysis, only the presence of depression on the HAM-D scale retained significant associations with MACCE risk (Table 5, Figure 2).

### 3.5. One-Year Kaplan–Meier Survival Curves Depending on Depression Incidence in BDI and HAM-D Scales, and Based on Reason for Hospitalization

**Figure 3:**
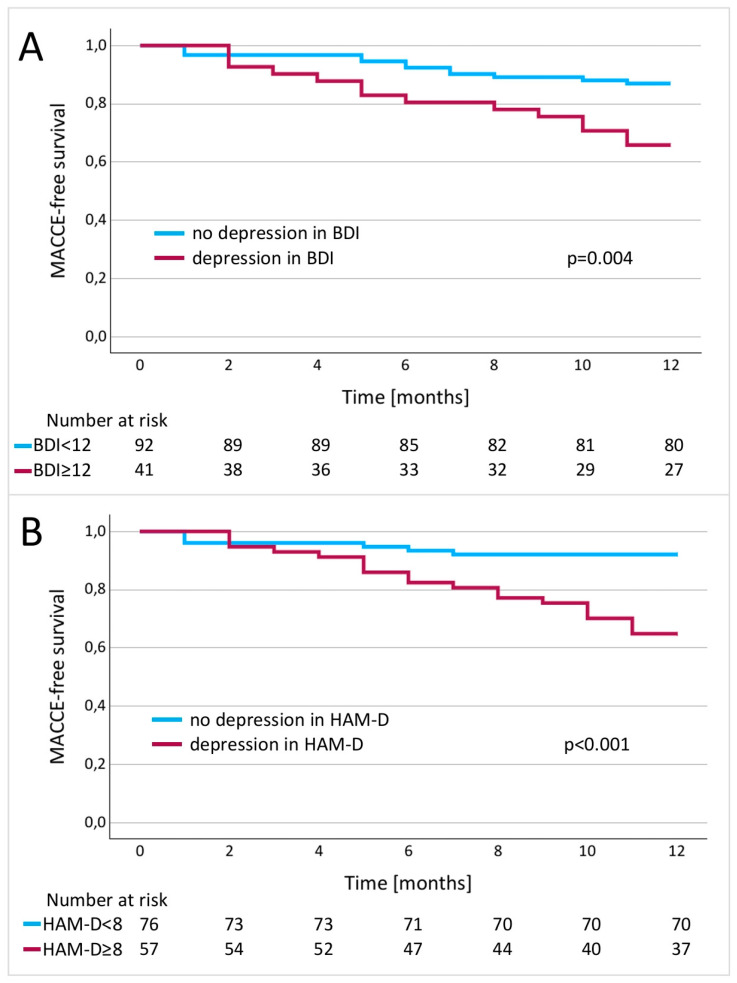
Kaplan–Meier curves for MACCE-free survival based on BDI (**A**) and HAM-D (**B**) results.

Kaplan–Meier MACCE-free survival curves at the 6-, and 12-month follow-ups were 95% and 84% for patients without depressive symptoms, compared to 85% and 65% for patients with depression according to the BDI scale (*p* = 0.004), and 94% and 91% vs. 82% and 64% according to the HAM-D scale (*p* < 0.001), respectively (Figure 3).

**Figure 4:**
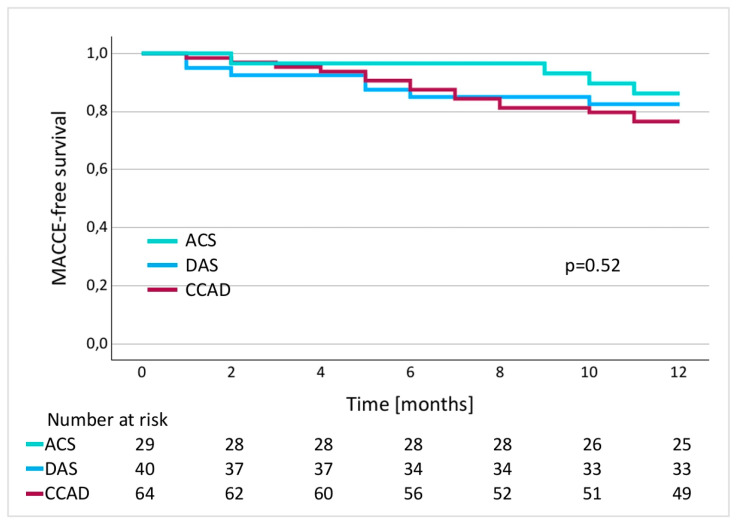
Kaplan–Meier curves for MACCE-free survival based on the reason for hospitalization.

Importantly, no differences were observed in terms of MACCE-free survival according to the cause of hospitalization and interventional treatment (Figure 4).

## 4. Discussion

This study reveals a high prevalence of depression among cardiac patients treated with invasive procedures, ranging from 31% to 43%, depending on the test performed. Furthermore, the study demonstrated an increased risk of MACCE among patients who underwent invasive cardiac procedures when depressive symptoms were present. In particular, the risk increases 3.6-fold when depression is identified using the BDI scale, and 4.9-fold using the HAM-D test. These findings are significant because depression has a relatively common occurrence among cardiovascular patients even considered to be well-managed. It can be noticed, both in patients with chronic cardiovascular diseases like DAS and CCAD, as well as in patients with acute cardiovascular disease, like the ACS [32]. Regretfully, ‘psyche’ tests are seldom performed in cardiovascular units. Despite the American Heart Association and the European Society of Cardiology recommendations, the screening, referral, and treatment of depression in patients with cardiovascular disease are very limited in cardiac departments [33]. Therefore, the integrated management of depression and cardiovascular disease is especially critical, as these conditions represent two of the leading contributors to disability-adjusted life years (DALYs) in high-income countries [34].

### 4.1. Depression Prevalence

Depression is estimated to affect approximately 18% to 31% of individuals with cardiovascular disease [35,36,37,38]; however, some studies indicate percentages as high as 79.1% [39]. Thus, our results are in line with previous observations. Approximately 20% of patients who sustain a myocardial infarction meet the criteria for a major depressive episode at the time of their cardiac event [40]. Similarly, at least as many patients with heart failure are depressed [41]. Anxiety and depression levels can be elevated in up to 43% of patients during the first 12 months after an acute cardiovascular event [42]. This indicates that depression is a dynamic process. In the study by Vatsa et al., depression was diagnosed in 38.8% of cardiac patients at baseline using a Patient Health Questionnaire 9 (PHQ9); however, a new depression episode was developed in an additional 15.9% at the one-year follow-up [43]. Therefore, conducting periodic assessments of patients’ depressive symptoms should be considered to facilitate a comprehensive and individualized approach to their care. In conclusion, our data, along with the above-mentioned data, suggest that patients with depression are commonly seen in cardiology departments, underlining the need for greater emphasis on identifying and addressing depression as a significant cardiovascular risk factor.

### 4.2. Depression Risk Factors

In our study, the results of the HAM-D scale differed with respect to some cardiovascular risk factors, while the BDI scale failed to show such associations. When using HAM-D, patients with scores indicative of depression were older, had lower eGFR, and exhibited a higher prevalence of diabetes, atrial fibrillation, and anticoagulant use.

The median age of participants in our study was 71 (65–75) years, thereby categorizing the majority as belonging to the elderly population. Patients with diagnosed depression on the HAM-D scale were significantly older than those without such a diagnosis (75 vs. 68 years, *p* < 0.001). The risk of depression appears to escalate with increasing patient age [44,45]. Some data suggest that over a third of older populations globally have depression [46,47]. Furthermore, in older adults, depression often remains undetected and untreated [48]. Therefore, in an aging population [25,26], additional attention should be paid to diagnosing depression, and individualized treatment should be administrated among those patients.

In our study, 26% of patients had atrial fibrillation and 28% were on permanent anticoagulation therapy. Both conditions were more common in depressed patients (respectively: 36.8% vs. 17.1%, *p* = 0.015 and 38.6% vs. 19.7%, *p* = 0.02). The prevalence of depression in patients with AF is estimated at 24% in the general population, and this rate increases to around 40% among older adults [49]. Depression has been shown to increase the risk of both the onset of new AF and the recurrence of episodes in patients with pre-existing AF [50,51,52]. Conversely, the presence of AF exacerbates depressive symptoms [53]. Furthermore, AF is described as more closely associated with depressive symptoms than coronary artery disease [54]. One contributing factor to the high prevalence of depression among patients with AF may be the necessity of long-term anticoagulant therapy [55]. Anticoagulation therapy, particularly when combined with dual antiplatelet therapy, is associated with increased mortality and higher rates of bleeding complications [56]. The fear of bleeding may impact even 60% of patients with AF [57]. Moreover, depression is associated with decreased adherence to anticoagulation therapy [58,59]. 

Diabetes was associated with a 2-fold increased prevalence of depressive symptoms in our study. It is excessively prevalent in the aging population, imposing additional risk of panvascular angiopathy, nephropathy, neuropathy, and stroke [60]. Studies suggest that depression occurs in about 20–35% of patients with diabetes [61,62,63,64,65] and is more common among patients with type 1 diabetes, compared to type 2 [66,67]. Interestingly, in patients with type 2 diabetes, the rate of depression was higher among those treated with insulin and oral medications compared to those taking only oral medications, and highest in patients treated with insulin alone [67], with odds ratio (OR): 1.59 (95%CI: 1.41–1.80, *p* < 0.001) [68]. The above observations are consistent with our results, in which insulin administration showed a three times higher depression rate as compared to non-insulin users diabetic patients (17.5% vs. 5.3%). The presence of type 2 diabetes significantly elevates the risk of major depression (HR: 1.61, 95%CI: 1.49–1.77]) [69]. The link between depression and diabetes is complex, including biological, psychological, and social factors [70]. Some studies suggest that one of the common biological origins of type 2 diabetes and depression may be the overactivation of innate immunity that leads to a cytokine-mediated inflammatory response, resulting in dysregulation of the hypothalamic–pituitary–adrenal axis [71,72]. In contrast, other studies emphasize the role of genetic [73], social [74], as well as environmental factors [75]. In conclusion, it is essential to consider the potential for depression when managing diabetic patients, particularly those undergoing insulin therapy.

In our study, the median eGFR was significantly lower in the group with depression on the HAM-D scale, compared to the non-depressed group (67 vs. 80 mL/min/1.73 m^2^, *p* = 0.002). The prevalence of depression among patients with chronic kidney disease is estimated between 20 and 30% [76,77,78,79]. However, some studies suggest that depression affects a significantly higher proportion of patients with kidney failure, with rates approaching nearly 100% [80,81]. The incidence of depression appears to increase with the degree of chronic kidney disease [82], reaching a maximum among patients on dialysis [76,83,84]. The presence of renal failure is associated with an increased risk of a depressive episode (HR: 1.36, 95%CI: 1.05–1.76) [85]. On the other hand, the presence of depression significantly increases the risk of kidney disease progression (HR: 1.38, 95%CI: 1.28–1.48), MACCE (HR: 1.22, 95%CI: 1.18–1.27), and all-cause mortality (HR: 1.41, 95%CI: 1.37–1.45) [86].

Some research data indicates that depression is significantly more prevalent among women than men [87]. The underlying causes of this disparity encompass not only biological factors but also differences in societal roles, representing a gender minority, low self-esteem, and gender-based violence [88]. In our study, the proportion of women to men in the non-depressed group, as assessed by HAM-D, is notably lower than in the depressed group, though this difference does not reach statistical significance. This is likely attributable to the limited size of the study group. An increase in the number of participants would probably reveal sex-related differences in the prevalence of depression. 

In summary, older age, atrial fibrillation, anticoagulant use, diabetes, and chronic kidney disease are all established risk factors for depression according to the HAM-D scale, but were not identified as such on the BDI scale. Given that each of these conditions is frequently observed in cardiac patients, an individualized assessment of depression risk factors should be an integral component of effective treatment strategies.

### 4.3. Depression and MACCE Risk

Cardiovascular interventions aim at the improvement of patients’ care, preferably symptom relief, prevention of adverse events, and life-expectancy prolongation [89,90,91,92]. However, an individual patient’s quality of life is not only attributed to the success deriving from the well-conducted cardiovascular procedure as such. The individual patient’s well-being is a much more complex phenomenon [93,94,95]. This study demonstrated that there are psychological factors equally decisive and important in patients’ recovery. Depression and anxiety experienced by the patient because of somatic cardiovascular disease and fear resulting from the interventional procedure had an impact on the mortality and cardiovascular outcomes. Attention should also be paid to less canonical albeit important factors, including molecular, inflammatory, and imaging workups that are gaining increasing attention [96,97,98]. They offer additional information on disease halting or progression [99,100,101]. 

MACCE is a composite endpoint commonly utilized in outcome evaluations within clinical trials. Its components vary depending on the study’s methodology; nevertheless, all-cause or cardiovascular death, myocardial infarction, and stroke can be singled out as constant elements. Other components used in studies include the need for revascularization treatment, or re-hospitalization for heart failure [102,103,104].

In a meta-analysis by Krittanawong et al. of 26 studies with a total of nearly 2 million patients, the presence of depression was associated with increased risk of stroke (HR: 1.13; 95%CI: 1.00–1.28), myocardial infarction (HR: 1.28, 95%CI: 1.14–1.45), congestive heart failure (HR: 1.04, 95%CI: 1.00–1.09), or any cardiovascular disease (HR: 1.16, 95%CI: 1.04–1.30). Depression was associated with increased risk of all-cause mortality (HR: 1.43, 95%CI: 1.27–1.60), cardiovascular disease mortality (HR: 1.44, 95%CI: 1.27–1.63), and congestive heart failure mortality (HR: 3.20, 95%CI: 1.29–7.94) [105]. However, the meta-analysis was limited to individuals with undiagnosed cardiovascular disease. For this reason, our study is relevant because it demonstrates depression prevalence and its impact on the outcomes in enormously high-cardiovascular-risk patients. In the present study, depression was associated with over a 3-fold risk increase for one-year adverse cardiovascular events. 

An interesting meta-analysis was presented by Zhang et al. In this meta-analysis, in patients who underwent percutaneous coronary intervention (PCI) for coronary artery disease, the presence of depression was associated with a higher risk of major cardiac events (MACEs) (HR: 1.89, 95%CI: 1.33–2.68), and all-cause mortality (HR: 1.71, 95%CI: 1.43–2.05) [106].

Dadkhah-Tirani et al. reported that depression diagnosed using BDI increased the risk of MACCE and re-hospitalization up to 2.045 times among patients after coronary angiography in a 12-month follow-up [107]. Moreover, one-year MACE-free survival was associated with depression severity, with rates of 54% for mild severity, 58% for moderate depression, and 39% for severe depression, compared to 74% for patients without depressive symptoms, *p* < 0.001.

Another study by Vatsa et al. on patients with CAD revealed that a higher PHQ9 score was associated with higher MACCE and HF hospitalization rates in a five-year follow-up, with each point increase on the scale (HR: 1.02, 95%CI: 1.00–1.03, *p* = 0.03) [43]. Moreover, for patients with persistent or increased PHQ-9 scores after one year of follow-up, the likelihood of developing MACCE was 96% higher (*p* = 0.04), compared to those with an improvement in PHQ9 test performance.

Thus, in our opinion, optimizing patient care by engaging additional diagnostic workups as well as a multidisciplinary approach is the right direction to provide an optimal quality of care to each individual patient.

### 4.4. Connection Between Cardiovascular Disease and Depression

Depression can deteriorate the natural course of cardiovascular disease, and cardiovascular disease can trigger depressive symptoms [108]. The links between depression and cardiovascular disease occur at many levels, including biological, psychological, social, and environmental factors. The shared pathogenesis of both diseases may involve the hypothalamic–pituitary–adrenal axis, genetic predispositions, immune system (inflammatory), autonomic dysfunction, serotonin pathways, microRNA, Omega-3 polyunsaturated fatty acids, and intestinal flora [109,110]. Nevertheless, behavioral and lifestyle factors should not be overlooked. Smoking, alcohol consumption, physical inactivity, and obesity are among the main factors more commonly found in patients with depression, all of which have detrimental effects on the cardiovascular system [111,112]. Moreover, depression is associated with an increased risk of nonadherence to medication, and consequently ineffective treatment [30,113]. Finally, depression is linked to an increased risk of social isolation and economic burden, both of which contribute to a poorer cardiovascular prognosis [4].

### 4.5. The Applicability of HAM-D and BDI Scales in Cardiovascular Patients

One of our findings is that the BDI score was more suitable when the cutoff point for depression was 11 points or more (AUC: 0.65, *p* = 0.015), despite a score of 12 points being assumed as indicative of depression. For the HAM-D scale, the cutoff point of eight or more points was consistent with the established scoring criteria. In our study, HAM-D identified depression in a significantly higher number of patients. Moreover, HAM-D was more effective than BDI in highlighting distinct risk factors that differentiate patients with depression from those without. The observed differences may be attributed to the distinct characteristics of the two scales. BDI is a self-reporting questionnaire, taking a few minutes to complete [114], although initially designed to be administered by trained interviewers [31]. HAM-D, on the other hand, is designed to be used by a healthcare professional [9]. The interview using HAM-D should typically take between 15 and 20 min [9], but the time may vary due to psychomotor retardation [1].

An interesting study by Seemüller et al. [115] examined the consistency and factor structure of BDI, HAM-D, and the Montgomery Asberg Depression Rating Scale. The findings suggest that self-reported and clinician-administered assessments are complementary rather than superfluous. Comprehensive depression diagnostics should therefore include multiple measures utilizing different questionnaires. A similar conclusion was made by Schneibel et al. suggesting that HAM-D and BDI should be considered complementary rather than redundant or competing instruments, as discrepancies between them may be linked to individual personality characteristics [116]. Another study by Tung et al. [117] compared HAM-D, the Hamilton Anxiety Rating Scale, BDI, and Zung’s Self-Rating Anxiety in terms of screening severe depression among patients with recurrent depression disorder. The authors concluded that BDI had the best diagnostic effect with the most optimal sensitivity and specificity, and cutoff values of all scales should vary depending on gender and age. Contrary to the above, the results of our study suggest that HAM-D is superior to BDI. Nonetheless, the above studies concern non-cardiac populations. Therefore, there remains uncertainty about which depression assessment tool is best suited for patients in cardiac units. The Cardiac Depression Scale is the only instrument designed to measure depression in cardiac patients [118]. Nevertheless, it is not often used in studies evaluating depression among patients with cardiovascular disease [4,119]. Although the 2021 ESC Guidelines on cardiovascular disease prevention in clinical practice recommend depression screening, no exact questionnaire is indicated, giving only the PHQ as an example [120]. The PHQ2 (2 items) and PHQ9 (9 items) are the most common clinical tests for assessing depression. They are easy to use, available in multiple languages and accessible through the public domain. For these reasons, they seem to be the best tools to screen for depression in CVD patients [4]. However, it is not possible to assess baseline symptom severity and to monitor subsequent improvement with antidepressant treatment [1]. González-Roz et al. [119] investigated the diagnostic accuracy of depression questionnaires for cardiac population, revealing that the BDI and the Hospital Anxiety and Depression Scale showed the best sensitivity and negative predictive values for detecting depression. The authors concluded that depression screening in cardiac patients should be conducted using the BDI. In case of limited time or resources, the PHQ-2 can be used initially, followed by the BDI if the PHQ-2 results are positive [120]. 

In conclusion, determining the optimal questionnaire for screening and assessing the severity of depression among cardiac patients remains challenging. The authors of this study recommend a comprehensive assessment of depressive symptoms using both self-reported and clinician-administered tools whenever feasible. Such an approach allows for a more appropriate, individualized assessment, leading to a more accurate diagnosis and, as a result, more effective treatment and improved prognosis.

### 4.6. Key Findings for Future Studies

Screening tools such as BDI and HAM-D are widely used and recognized instruments in daily prevention, assessment of depression severity, and monitoring the course of its treatment. The study results indicate the significant role of prevention, early diagnosis, and, if necessary, the implementation of depression treatment in patients after cardiac incidents. To this end, it is worth deepening this research in a multicenter model. It is also advisable to introduce psychoeducational work among patients and medical staff, including both doctors and nurses, at this stage. Patients can independently monitor their well-being using the BDI tool, while medical staff can regularly assess depression severity with HAM-D. Whenever results indicate mild, moderate, or severe depression, it is essential to deepen the diagnosis by conducting a comprehensive interview and considering treatment options, particularly offering psychotherapeutic support. Collaboration between psychiatrists and cardiologists will be crucial in developing effective educational programs that build awareness within the medical community.

### 4.7. Study Limitations

One of the most obvious limitations of our study is the small number of study participants; however, this is our preliminary report. Also, BDI and HAM-D are not the most used scales; nonetheless, it opens the way for follow-up and reassessment of depression level after the cardiac intervention. The results of this study should be interpreted with caution. Further research is necessary to evaluate the relationship between scores on the BDI and the HAM-D and the associated risk of MACCE.

## 5. Conclusions

Depression is a common finding in cardiovascular patients, and it is a strong risk factor for one-year mortality and adverse cardiovascular events. Therefore, we believe that common guidelines should be elaborated between relevant psychiatry and cardiology scientific societies to enhance the care of patients with cardiovascular disease and depression.

## Figures and Tables

**Table 1:** Baseline study group characteristics.

	Patients with Normal BDI Score n = 92	Patients with BDI Score Indicating Depression n = 41	*p* Value	Patients with Normal HAM-D Score n = 76	Patients with HAM-D Score Indicating Depression n = 57	*p* Value
Age [years]	70 (63–76)	73 (67–79)	0.16	68 (62–74)	75 (69–80)	<0.001
Males	64 (69.6%)	25 (61%)	0.43	56 (73.7%)	33 (57.9%)	0.06
Females	28 (30.4%)	16 (39%)		20 (26.3%)	24 (42.1%)	
BMI [kg/m^2^]	27 (25–31)	28 (25–34)	0.57	27 (25–32)	28 (25–31)	0.85
Reason for hospitalization:						
CCAD	48 (52.2%)	16 (39%)	0.39	43 (56.6%)	21 (36.8%)	0.044
ACS	18 (19.6%)	11 (26.8%)		16 (21.1%)	13 (22.8%)	
DAS	26 (28.3%)	14 (34.1%)		17 (22.4%)	23 (40.4%)	
Hypertension	83 (90.2%)	35 (85.4%)	0.55	67 (88.2%)	51 (89.5%)	>0.99
Hyperlipidemia	82 (89.1%)	36 (87.8%)	0.77	66 (86.8%)	52 (91.2%)	0.58
Diabetes	38 (41.3%)	24 (58.5%)	0.09	24 (31.6%)	38 (66.7%)	<0.001
Diabetes treated with oral drugs	31 (33.7%)	17 (41.5%)	0.44	20 (26.3%)	28 (49.1%)	0.01
Diabetes treated with insulin	7 (7.6%)	7 (17.1%)	0.13	4 (5.3%)	10 (17.5%)	0.042
Current smoking	47 (51.1%)	19 (46.3%)	0.71	39 (51.3%)	27 (47.4%)	0.73
COPD/asthma	10 (10.9%)	4 (9.8%)	>0.99	6 (7.9%)	8 (14%)	0.39
Creatinine [μmol/L]	84 (75–103)	91 (77–122)	0.19	83 (74–105)	91 (76–111)	0.12
eGFR [mL/min/1.73 m^2^]	77 (59–88)	72 (43–87)	0.08	80 (65–90)	67 (48–80)	0.002
TSH [μU/mL]	1.6 (0.8–2.8)	1.8 (1–2.9)	0.29	1.8 (1–3.1)	1.5 (0.8–2.6)	0.18
Thyroid disease	14 (16.9%)	8 (22.2%)	0.61	11 (15.9%)	11 (22%)	0.48
Atrial fibrillation	22 (23.9%)	12 (29.3%)	0.53	13 (17.1%)	21 (36.8%)	0.015
Anticoagulants	26 (28.3%)	11 (26.8%)	>0.99	15 (19.7%)	22 (38.6%)	0.02
NOAC	23 (25%)	10 (24.4%)	>0.99	13 (17.1%)	20 (35.1%)	0.025
VKA	3 (3.3%)	1 (2.4%)	>0.99	2 (2.6%)	2 (3.5%)	>0.99
Antiplatelets	73 (79.3%)	34 (82.9%)	0.65	63 (82.9%)	44 (77.2%)	0.51
ASA	67 (72.8%)	29 (70.7%)	0.84	58 (76.3%)	38 (66.7%)	0.25
P2Y12 inhibitors	48 (52.2%)	22 (53.7%)	>0.99	44 (57.9%)	26 (45.6%)	0.22

ACS—acute coronary syndrome; ASA—acetylsalicylic acid; BDI—Beck Depression Inventory; BMI—body mass index; CCAD—chronic coronary artery disease; COPD—chronic obstructive pulmonary disease; DAS—degenerative aortic valve stenosis; eGFR—estimated glomerular filtration rate; HAM-D—Hamilton Depression Rating Scale; NOAC—non–vitamin K oral anticoagulants; TSH—thyroid stimulating hormone; VKA—vitamin K antagonists.

**Table 2:** Characteristics of patients by severity of depression in BDI and HAM-D.

	Depression Severity According to BDI				Depression Severity According to HAM-D				
	Normal n = 92	Mild n = 39	Moderate n = 2	*p* Value	Normal n = 76	Mild n = 32	Moderate n = 16	Severe n = 9	*p* Value
Age [years]	70 (63–76)	73 (68–79)	69	0.34	68 (62–74)	76 (70–82)	71 (67–77)	78 (70–82)	<0.001 *
Males	64 (69.6%)	23 (59%)	2 (100%)	0.35	56 (73.7%)	18 (56.3%)	10 (62.5%)	5 (55.6%)	0.24
Females	28 (30.4%)	16 (41%)	0 (0%)		20 (26.3%)	14 (43.8%)	6 (37.5%)	4 (44.4%)	
BMI [kg/m^2^]	27 (25–31)	28 (25–32)	31	0.61	27 (25–32)	27 (25–29)	28 (24–35)	28 (24–32)	0.96
Reason for hospitalization:									
CCAD	48 (52.2%)	14 (35.9%)	2 (100%)	0.29	43 (56.6%)	14 (43.8%)	3 (18.8%)	4 (44.4%)	0.11
ACS	18 (19.6%)	11 (28.2%)	0 (0%)		16 (21.1%)	7 (21.9%)	5 (31.1%)	1 (11.1%)	
DAS	26 (28.3%)	14 (35.9%)	0 (0%)		17 (22.4%)	11 (34.4%)	8 (50%)	4 (44.4%)	
Hypertension	83 (90.2%)	33 (84.6%)	2 (100%)	0.51	67 (88.2%)	30 (93.8%)	12 (75%)	9 (100%)	0.21
Hyperlipidemia	82 (89.1%)	34 (87.2%)	2 (100%)	0.82	66 (86.8%)	30 (93.8%)	14 (87.5%)	8 (88.9%)	0.79
Diabetes	38 (41.3%)	24 (61.5%)	0 (0%)	0.024	24 (31.6%)	21 (65.6%)	10 (62.5%)	7 (77.8%)	<0.001
Diabetes treated with oral drugs	31 (33.7%)	17 (43.6%)	0 (0%)	0.37	20 (26.3%)	16 (50%)	5 (31.3%)	7 (77.8%)	0.005
Diabetes treated with insulin	7 (7.6%)	7 (17.9%)	0 (0%)	0.25	4 (5.3%)	5 (15.6%)	5 (31.3%)	0 (0%)	0.013
Current smoking	47 (51.1%)	18 (46.2%)	1 (50%)	0.85	39 (51.3%)	16 (50%)	8 (50%)	3 (33.3%)	0.83
COPD/asthma	10 (10.9%)	4 (10.3%)	0 (0%)	>0.99	6 (7.9%)	6 (18.8%)	0 (0%)	2 (22.2%)	0.10
Creatinine [µmol/L]	84 (75–103)	91 (78–122)	79	0.25	83 (74–105)	93 (82–109)	84 (68–92)	111 (73–153)	0.11
eGFR [mL/min/1.73 m^2^]	77 (59–88)	71 (40–86)	86	0.09	80 (65–90)	59 (46–78)	78 (57–89)	48 (35–79)	0.001 **
TSH [μIU/mL]	1.6 (0.8–2.8)	1.8 (1–2.8)	2.9	0.43	1.5 (0.8–2.6)	1.8 (1.3–3.4)	1.6 (0.6–2.5)	1.5 (1–3.5)	0.32
Thyroid disease	14 (16.9%)	8 (22.9%)	0 (0%)	0.55	11 (15.9%)	8 (28.6%)	2 (14.3%)	1 (12.5%)	0.51
Atrial fibrillation	22 (23.9%)	11 (28.2%)	1 (50%)	0.47	13 (17.1%)	12 (37.5%)	4 (25%)	5 (55.6%)	0.021
Anticoagulants	26 (28.3%)	10 (25.6%)	1 (50%)	0.69	15 (19.7%)	15 (46.9%)	4 (25%)	3 (33.3%)	0.035
NOAC	23 (25%)	9 (23.1%)	1 (50%)	0.65	13 (17.1%)	14 (43.8%)	4 (25%)	2 (22.2%)	0.037
VKA	3 (3.3%)	1 (2.6%)	0 (0%)	>0.99	2 (2.6%)	1 (3.1%)	0 (0%)	1 (11.1%)	0.40
Antiplatelets	73 (79.3%)	33 (84.6%)	1 (50%)	0.33	63 (82.9%)	24 (75%)	13 (81.3%)	7 (77.8%)	0.76
ASA	67 (72.8%)	28 (71.8%)	1 (50%)	0.75	58 (76.3%)	21 (65.6%)	11 (68.8%)	6 (66.7%)	0.64
P2Y12 inhibitors	48 (52.2%)	21 (53.8%)	1 (50%)	>0.99	44 (57.9%)	15 (46.9%)	6 (37.5%)	5 (55.6%)	0.43

*—post hoc analysis: normal vs. mild, *p* = 0.001; normal vs. severe, *p* = 0.004. **—post hoc analysis: normal vs. mild, *p* = 0.005. ACS—acute coronary syndrome; ASA—acetylsalicylic acid; BDI—Beck Depression Inventory; BMI—body mass index; CCAD—chronic coronary artery disease; COPD—chronic obstructive pulmonary disease; DAS—degenerative aortic valve stenosis; eGFR—estimated glomerular filtration rate; HAM-D—Hamilton Depression Rating Scale; NOAC—non–vitamin K oral anticoagulants; TSH—thyroid stimulating hormone; VKA—vitamin K antagonists.

**Table 3:** Incidence of major adverse cardiac and cerebrovascular events, all-cause death, cardiovascular death, myocardial infarction, ischemic stroke, hospitalization for acute heart failure, and dementia depending on the depression presence in BDI and HAM-D at 12-month follow-up.

	Patients with Normal BDI Score n = 92	Patients with BDI Score Indicating Depression n = 41	*p* Value	Patients with Normal HAM-D Score n = 76	Patients with HAM-D Score Indicating Depression n = 57	*p* Value
MACCE	12 (13%)	14 (34.1%)	0.008	6 (7.9%)	20 (35.1%)	<0.001
All-cause death	4 (4.3%)	5 (12.2%)	0.13	2 (2.6%)	7 (12.3%)	0.038
CVD	3 (3.3%)	2 (4.9%)	0.64	1 (1.3%)	4 (7%)	0.16
Myocardial infarction	1 (1.1%)	1 (2.4%)	0.52	1 (1.3%)	1 (1.8%)	>0.99
Ischemic stroke	3 (3.3%)	1 (2.4%)	>0.99	3 (3.9%)	1 (1.8%)	0.64
AHF	4 (4.3%)	8 (19.5%)	0.008	0 (0%)	12 (21.1%)	<0.001
New dementia	2 (2.2%)	1 (2.4%)	>0.99	1 (1.3%)	2 (3.5%)	0.58

AHF—acute heart failure; BDI—Beck Depression Inventory; CVD—cardiovascular death; HAM-D—Hamilton Depression Rating Scale; MACCE—major adverse cardiac and cerebrovascular event.

**Table 4:** ROC analysis and cutoff points for the occurrence of a composite endpoint.

	AUC	95%CI	*p* Value	Cutoff Point	Sensitivity	Specificity	Accuracy
BDI	0.65	0.53–0.77	0.015	≥11	0.62	0.74	0.71
HAM-D	0.73	0.62–0.84	<0.001	≥8	0.77	0.65	0.68
Age	0.57	0.45–0.69	0.24	≥68	0.81	0.38	0.47
BMI	0.53	0.4–0.66	0.70	≥34.55	0.25	0.87	0.77
Creatinine	0.57	0.44–0.7	0.30	≥130	0.23	0.92	0.78
eGFR	0.58	0.45–0.72	0.21	≤66	0.54	0.69	0.66
TSH	0.53	0.39–0.66	0.71	≥3.21	0.29	0.85	0.74

AUC—area under the curve; BDI—Beck Depression Inventory; BMI—body mass index; CI—confidence interval; eGFR—estimated glomerular filtration rate; HAM-D—Hamilton Depression Rating Scale; ROC—receiver operating characteristic; TSH—thyroid stimulating hormone.

**Table 5:** The univariate and multivariate Cox proportional hazard analysis of factors associated with MACCE.

		Univariate Cox HR (95%CI); *p* Value	Multivariate Cox HR (95%CI); *p* Value
BDI score ≥ 11 points		3.61 (1.64–8.0), 0.001	2.06 (0.88–4.83), 0.10
HAM-D score ≥ 8 points		4.91 (1.97–12.24), <0.001	3.08 (1.18–8.08), 0.022
Antidepressants use		1.01 (0.3–3.36), 0.99	
Age [years]		1.02 (0.97–1.06), 0.46	
Male sex		0.76 (0.35–1.68), 0.50	
BMI [kg/m^2^]		1.03 (0.96–1.1), 0.38	
Reason for hospitalization	CCAD	1.51 (0.69–3.29), 0.30	
	ACS	0.61 (0.21–1.76), 0.36	
	DAS	0.88 (0.37–2.09), 0.77	
Hypertension		0.96 (0.29–3.18), 0.94	
Hypercholesterolaemia		1.51 (0.36–6.4), 0.57	
Diabetes		1.98 (0.90–4.37), 0.09	
Diabetes treated with oral drugs		1.13 (0.51–2.49), 0.76	
Diabetes on insulin		3.02 (1.21–7.53), 0.018	2.2 (0.86–5.61), 0.10
Current smoking		1.42 (0.65–3.09), 0.38	
COPD/asthma		2.07 (0.78–5.48), 0.15	
Creatinine ≥ 130 μmol/L		2.85 (1.14–7.12), 0.024	2.03 (0.77–5.37), 0.15
eGFR [mL/min/1.73 m^2^]		0.98 (0.97–1), 0.07	
TSH [µU/mL]		1.04 (0.8–1.35), 0.79	
Thyroid disease		0.97 (0.33–2.86), 0.95	
Prior myocardial infarction		1.69 (0.78–3.67), 0.19	
Prior stroke		2.19 (0.83–5.82), 0.12	
Atrial fibrillation		2.71 (1.25–5.87), 0.011	1.85 (0.46–7.52), 0.39
Anticoagulants use (NOAC or VKA)		2.38 (1.1–5.14), 0.028	1.11 (0.28–4.39), 0.89
Antiplatelet therapy		0.78 (0.31–1.95), 0.60	
ASA		0.86 (0.37–1.97), 0.71	
P2Y12 inhibitors		0.9 (0.42–1.94), 0.79	

ACS—acute coronary syndrome; ASA—acetylsalicylic acid; BDI—Beck Depression Inventory; BMI—body mass index; CCAD—chronic coronary artery disease; CI—confidence interval; COPD—chronic obstructive pulmonary disease; DAS—degenerative aortic valve stenosis; eGFR—estimated glomerular filtration rate; HAM-D—Hamilton Depression Rating Scale; HR—hazard ratio; MACCE—major adverse cardiac and cerebrovascular event; NOAC—non–vitamin K oral anticoagulants; TSH—thyroid stimulating hormone; VKA—vitamin K antagonists.

## Data Availability

The raw data supporting the conclusions of this article will be made available by the authors on request.
